# Inaccuracy of the log‐rank approximation in cancer data analysis

**DOI:** 10.15252/msb.20188754

**Published:** 2019-08-12

**Authors:** Nimrod Rappoport, Ron Shamir

**Affiliations:** ^1^ Blavatnik School of Computer Science Tel Aviv University Tel Aviv Israel

## Abstract

The log‐rank test statistic is very broadly used in biology. Unfortunately, *P*‐values based on the popular chi‐square approximation are often inaccurate and can be misleading.

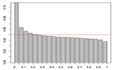

Comparing survival patterns between groups of individuals is ubiquitous in biomedical research. A significant difference in survival can show the efficacy of a drug or the biological relevance of a biomarker. In cancer research, clustering of patient profiles is used to discover disease subtypes (Prasad *et al*, 2016), and a significant difference in survival between clusters is usually considered a strong indication for a clustering algorithm's merit (Gabasova *et al*, 2017; Argelaguet *et al*, 2018). In these settings, the standard means to compare survival between groups of patients is the log‐rank test (Hosmer *et al*, 2008). We refer here to the conditional version of the test (see Appendix).

The log‐rank test is very broadly used. A Google Scholar search for “logrank test statistic” identifies > 22,000 citations, and a PubMed search in titles or abstracts for “logrank” or “log‐rank” identifies > 30,000 papers, and 3,357 published in 2018 alone. The real number of studies that use this test is likely even higher. The *P*‐value of the log‐rank test statistic is commonly approximated by the chi‐square distribution. We show here that in important contexts that approximation is poor and can be misleading.

The chi‐square approximation provides a good fit when there are a large number of events in each patient group and the group sizes are balanced. Heinze *et al* (2003) and Wang *et al* (2010) developed exact permutation tests that condition on the observed follow‐up in each group. While they showed that the asymptotic log‐rank test is inaccurate, the extent of this inaccuracy in practice, for real modern datasets that contain hundreds of patients and more than two clusters, is unclear.

We have recently benchmarked nine methods for clustering multi‐omic data across ten cancer cohorts from TCGA (The Cancer Genome Atlas Network, 2008; Rappoport & Shamir, 2018). Since survival information was available for the patients, we used the log‐rank test chi‐square approximation to evaluate each solution. In addition, we implemented the exact test developed by Heinze *et al* (2003) for more than two groups. We validated on simulated data that the implementation preserves the false‐positive rate better than the asymptotic version (see Appendix), and used the implementation to compute the exact test's *P*‐value (EP) of the log‐rank score for each solution on each cancer cohort. The results (Fig 1A) show large gaps between the EP and asymptotic *P*‐value (AP). In fact, the APs for 48 out of the 90 clustering solutions were not within their 95% confidence intervals constructed using the permutation test. This inaccuracy was exacerbated for small *P*‐values: 30 out of the 37 significant APs (≤ 0.05) did not fall within their 95% confidence intervals. In all these cases, the EPs were higher (less significant). In 17 out of the 37, the difference between the EP and the AP was at least 2‐fold. Three of the 37 cases reported as significant according to the asymptotic approximation (8%) were actually not significant according to the permutation tests.

**Figure 1:**
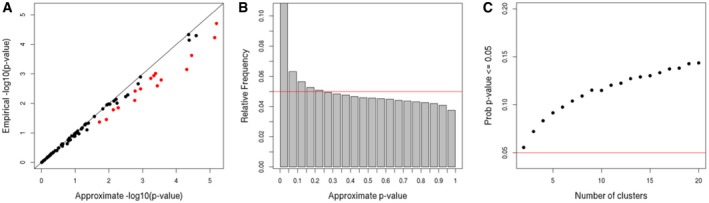
Asymptotic *P*‐values (APs) compared to *P*‐values based on permutation tests (EPs) (A) APs and EPs for clustering solutions of nine algorithms over ten cancer datasets. Red dots: 2AP < EP. MCCA's solution on KIRC is omitted. Confidence intervals for the EPs are small such that they are contained in the dots. (B) Distribution of APs across permutations of MCCA's solution on KIRC dataset. The red line represents the expected theoretical distribution. (C) The probability to observe AP ≤ 0.05 in random clustering solutions with different number of clusters on the breast TCGA dataset (see text).

Some asymptotic results were rather extreme. The MCCA method (Witten & Tibshirani, 2009) on the KIRC cancer dataset gave a clustering solution that obtained AP < 2e‐16, but EP = 6.8e‐5. The distribution of the APs over one million permutations of the KIRC cluster labels is shown in Fig 1B. By definition, that distribution should be uniform under the null hypothesis. However, 10.9% of the permutations received an AP ≤ 0.05.

We performed an additional test using the breast cancer dataset, which contains 621 patients. For each number *k* of clusters from 2 to 20, we partitioned the samples at random into *k* − 1 clusters of 10 patients and one large cluster with all other patients, and computed the APs. We repeated the process for many random permutations of the patient labels and calculated the fraction of permutations with AP ≤ 0.05 (see Appendix). The results are shown in Fig 1C. In spite of the large size of the breast cancer dataset, the probability to report a clustering as significant was markedly higher than 0.05 and increased as the number of clusters *k* increased. For *k* = 4, a common number of clusters for breast cancer datasets, the probability for AP ≤ 0.05 was already > 0.08.

How common is the use of the asymptotics in software tools? The R “survival” package, the Python “lifelines” package, SPSS, SAS and Stata all use the asymptotic test and report the same *P*‐values. While several packages do implement non‐asymptotic tests (see Appendix), they are less widely used. We conclude that the vast majority of the studies that perform the log‐rank test use the asymptotic *P*‐value.

A systematic search for cases where the use of the asymptotic test led to wrong conclusions is challenging: most studies do not publish survival data, and these data have no standard format. However, we were able to find recent cases where the test led to wrong or overstated reports. Joachim *et al* (2018) reported that use of the chemotherapeutic agent Topotecan resulted in a significant survival benefit in a murine model of endotoxemia. While the log‐rank AP was 0.042 for the presented data, the EP was actually 0.059. Gabasova *et al* (2017) developed a novel method for multi‐omic clustering and used it to cluster breast cancer data. The authors reported a *P*‐value of 0.038. As the version of log‐rank used was not specified, and the clustering solution was not provided, we could not calculate the EP. Instead, we permuted the group labels a large number of times, and for each permutation computed the AP of the conditional log‐rank, which is the more appropriate version to use in this scenario (see Appendix). For 13.5% of the permutations, the computed AP was ≤ 0.038, which shows that reporting the AP is not sufficient in this case to show a clustering solution's merit. Hence, erroneous significance conclusions due to the use of AP occur both in biomedical research and in algorithm development. Overstatement of significance is likely even more common.

The difference between asymptotic and exact tests is not unique to the log‐rank test. Rather, it is important for all statistical tests that rely on asymptotics, when sample size is small. In the log‐rank test, inaccuracy is not affected only by the sample size, but also by the number of events within each group, and by imbalance in the group sizes. In some other statistical tests, there is higher community awareness of inaccuracies. For example, the R implementation of the chi‐square test for independence issues warnings when used with small sample sizes. Such awareness should be raised for all asymptotic statistical tests.

Aside from the inaccuracy caused by using the asymptotic test, there are additional factors that one should consider when using the log‐rank test. The null hypothesis for the test with multiple groups is that the survival function is the same for all groups. The test will therefore reject the null hypothesis even in cases where only a single group differs from the others. Another factor to consider is that the test has low power when the different survival functions cross one another. Analysis of differential survival for a clustering solution should therefore be accompanied by visualizing the Kaplan–Meier curve, and not by solely reporting the log‐rank *P*‐value, whether it is asymptotic or exact.

The log‐rank test is widely used to compare survival of different patient groups and to assess disease subtyping. It is perhaps the leading evaluation criterion that guides development of new computational methods for clustering patients. For large datasets with many events in each group, the asymptotic log‐rank test computes an accurate *P*‐value. However, our results show that *P*‐values based on the chi‐square approximation are highly inaccurate in evaluating clustering solutions of popular methods on real cancer datasets. It is therefore essential that future analyses compute and report *P*‐values using exact tests.

## Author contributions

NR and RS conceived the project and wrote the manuscript. NR performed the analysis. RS supervised the project.

## Supporting information



Appendix

## Data Availability

TCGA data after preprocessing for all cancer types are available here: http://acgt.cs.tau.ac.il/multi_omic_benchmark/download.html. Code to reproduce the analyses presented in this paper, and our implementation of the permutation‐based log‐rank test for more than two groups, are available in GitHub: https://github.com/Shamir-Lab/Logrank-Inaccuracies/tree/master.
