# Supplementary material for: Inaccuracy of the log‐rank approximation in cancer data analysis
Source: Mol Syst Biol. 2019 Aug 12;15(8):e8754. doi: 10.15252/msb.20188754 (PMC6690426; doi:10.15252/msb.20188754)
Supplement: Supplementary file 1 — Appendix [file MSB-15-e8754-s001.pdf]

Appendix

Inaccuracy of the log-rank approximation in cancer data analysis

Nimrod Rappoport and Ron Shamir*

The Blavatnik School of Computer Science, Tel Aviv University, Tel Aviv 69978, Israel

*To whom correspondence should be addressed. Tel: +972 3 640 5383; Fax: +972 3 640 5384;

Email: rshamir@tau.ac.il (RS)

Appendix Table S1: Page 5.

Appendix Table S2: Page 6.

Figure S1 and Legend: Page 8.

Survey of the log-rank test

The log-rank test is commonly used to compare survival between several groups of individuals, where the observations can be censored.

Following the notation in Heinze et al (2003), we assume n independent individuals that are divided into r+1 group. Denote by r_i, x_i, y_i the group, event (death) time and follow-up time of individual i. The observed values for individual i are $t_i = \min(x_i, y_i)$, the observed survival time, and $\delta_i = I_{x_i \leq y_i}$, its censoring status, where I is the indicator function. The event times in each group j are assumed to be sampled from a distribution function T_j, and the follow-up times to be sampled from F_j. Log-rank tests the null hypothesis that $T_j = T_{j'}$ for all j, j'.

Two different versions of the test exist, and they are both referred to as "log-rank". In the version introduced in Peto and Peto (1972), it is further assumed that $F_j = F_{j'}$ for all j, j', while in the version introduced by Mantel (1966) the follow-up distributions are not assumed to be identical. We refer to the first version of the test as the permutational log-rank, and to the second version as the conditional log-rank.

We will use the following notation:

q - the number of time points at which an event was observed.
N_{jh} - the number of individuals at risk in group j at time h.
N_h - the total number of individuals at risk at time h.
M_{jh} - the number of events in group j at time h.
M_h - the total number of events at time h.
$A_{jh} = \frac{N_{jh}}{N_h}$ - the relative number of individuals in group j at time h.
$S_j = \Sigma_{h=1}^{q} M_{jh}$ - the total number of events in group j.
$E_j = \Sigma_{h=1}^{q} M_h A_{jh}$ - the expected number of events in group j.

$$V_{jk} = \Sigma_{h=1}^{q} \left(\frac{M_h (N_h - M_h)}{N_h - 1} A_{jh} (I_{j=k} - A_{kh}) \right)$$

Let $S = (S_1, \ldots S_r)$, $E = (E_1, \ldots E_r)$ and V the r x r matrix with V_{jk} as entries.

In the conditional log-rank, the test statistic is $(S - E)V^{-1}(S - E)^t$. Its asymptotic distribution under the null hypothesis is χ^2 with r degrees of freedom.

In the permutational log-rank, the test statistic is $(S - E)Var(S - E)^{-1}(S - E)^t$. Its asymptotic distribution under the null hypothesis is χ^2 with r degrees of freedom as well.

Asymptotically, the distributions of the two test statistics are identical (Mantel, 1985). However, for small sample sizes the distributions differ. As mentioned previously, the permutational test assumes that $F_j = F_{j'}$ for all j, j'. It is the same null hypothesis tested by randomly permuting individuals between groups while conditioning on the group sizes. This hypothesis can therefore be tested by enumerating over such permutations, giving an exact version of the test (Peto and Peto, 1972). Vandin et al (2015) recently developed an efficient algorithm to test this hypothesis.

For the conditional test, Heinze et al (2003) and Wang et al (2010) developed permutation-based versions of the test (Wang et al for the two group case). Heinze et al (2003) developed two tests, one that conditions on the observed risk set tables (termed ECR), and another

that conditions on observed follow-up (termed ECF). Simulation experiments showed that only the version that conditions on observed follow-up maintains the desired false-positive rate.

The conditional log-rank is more suitable for comparing the survival functions between clusters discovered in retrospective analysis on cancer data, like we perform here, since the follow-up may be affected by factors that are associated with the discovered clusters. Additional discussion on the choice of the test's variance is available in Brown (1984) and Mantel (1985).
We therefore use the permutation-based conditional test that conditions on observed follow-up (ECF), as presented in Heinze et al (2003), and refer to its resulting p-value as the exact test's p-value (EP). We implemented the test, as an implementation of it was only available for the case of two groups. Briefly, the test permutes the (t_i, δ_i) values between the patients. For each individual, if its permuted event time is censored, it is imputed from an empirical Kaplan-Meier estimator of the global survival function. Likewise, if its original follow-up time is censored, it is imputed from an empirical Kaplan-Meier estimator of its group's follow-up function. Refer to Heinze et al (2003) for full details.

Implementations of the log-rank test

Here, "empirical" tests are permutation-based that do not exhaust all options, while "exact" ones do enumerate all permutations.

R:
function survdiff in package survival – asymptotic conditional.
function permHeinze in package PermGS – empirical conditional (only for two groups).
function perm.test in package exactRankTests – exact and empirical permutational.
function logrank_test in package coin – exact, empirical (approximate) and asymptotic permutational.

Python:
function multivariate_logrank_test in package lifelines – asymptotic conditional.

SAS:
function LIFETEST – asymptotic conditional.
macro XLINRANK – empirical conditional.
Available in: https://cemsiis.meduniwien.ac.at/en/kb/science-research/software/statistical-software/xlinrank/

SPSS: Log rank test statistic – asymptotic conditional.

Stata: Log rank test statistic – asymptotic conditional.

StatXact: exact permutational.

Windows binary: XLINRANK – empirical conditional.
Available in: https://cemsiis.meduniwien.ac.at/en/kb/science-research/software/statistical-software/xlinrank/

Simulation studies

To test our implementation of the Heinze method, we performed size (false positive rate) analysis and power analysis. In each simulation, the data were generated in a fashion similar to Heinze et al (2003). Given the number of groups r+1 and a vector of group sizes S of length r+1, we assumed an accrual follow-up time that has uniform distribution between 12 and 60. We simulated follow-up times due to loss of contact, from an exponential distribution for each group with parameter vector γ_1 of length r+1, such that the rate used for group j is $\gamma_1[i]$. Similarly, we simulated survival times from an exponential distribution with parameter vector γ_2.

For individual i in group j(i) its accrual follow-up $u_i \sim Unif[12, 60]$, its contact follow-up $v_i \sim Exp(\gamma_1[i])$ and its survival time $x_i \sim Exp(\gamma_2[i])$. Its observed value is then set as $t_i = \min(u_i, v_i, x_i)$ and $\delta_i = 1$ if $x_i \leq \min(u_i, v_i)$.

For both the size and power analyses, we performed 13000 simulations for scenario, and report the fraction of simulations with p-value ≤ 0.05. We report this number for our implementation of the permutation-based method from Heinze et al (2003), and for the asymptotic conditional log-rank test as implemented in the survdiff function in the R package survival. For the Heinze et. al implementation, we used 1000 random permutations to assess the p-value.

The results of the size simulations are summarized in Table S1. All experiments here were done using a survival vector or length r+1 that is equal to (0.04, …, 0.04). That is, under the null hypothesis of equal survival functions. We performed 13000 simulations for each settings, such that the 99% confidence interval is less than 0.01 for a true effect size of 0.05 and the standard deviation for the estimator is < 0.02.

Table S1 – False Positive Rate Analysis

Simulation Number	S	γ_1 (follow-up)	% of permutations with $p \leq 0.05$	
			Exact	Asymptotic
1	(5, 5)	(0, 0)	0.047	0.065
2	(100, 100)	(0.04, 0.04)	0.051	0.053
3	(5, 5)	(0, 0.04)	0.042	0.058
4	(100, 100)	(0, 0.04)	0.049	0.050
5	(100, 100, 100, 100)	(0.04, 0.04, 0.04, 0.04)	0.049	0.050
6	(20, 70, 130, 180)	(0.04, 0.04, 0.04, 0.04)	0.052	0.057
7	(100, 100, 100, 100)	(0, 0, 0.04, 0.04)	0.048	0.051
8	(20, 70, 130, 180)	(0.08, 0.04, 0.04, 0)	0.049	0.054
9	(20, 70, 130, 180)	(0, 0.04, 0.04, 0.08)	0.052	0.056
10	(36, 40, 16, 43, 72, 134)	Sampled from a [0, 0.04] uniform distribution per group	0.054	0.061
11	(63, 58, 40, 31, 29, 25, 20, 19, 16, 10, 9, 9, 7, 5, 4, 3)	Sampled from a [0, 0.04] uniform distribution per group	0.049	0.115

Table S1 shows that the asymptotic test failed to maintain the false positive rate in several simulations, both with a low number of samples (simulations 1 and 3) and high number of samples, but with imbalanced groups sizes (simulations 6, 9, 10, 11). The group sizes in simulations 10 and 11 are taken from MKL's solution on lung cancer and MCCA's solution on kidney, respectively. These simulations show the inflated p-values that can be reported by the asymptotic test on real clustering solutions.

The results of the power simulations are summarized in Table S2. All experiments here were done using a survival vector or length r+1 where not all groups have the same event rate. That is, under the alternative hypothesis of unequal survival functions. We performed 13000 simulations for each settings, such that the 99% confidence interval is less than 0.01 for a true effect size of 0.05 and the standard deviation for the estimator is < 0.02.

Table S2 – Power Analysis

Simulation Number	S	γ_1 (follow-up)	γ_2 (event)	% of permutations with $p \leq 0.05$	
				Exact	Asymptotic
1	(5, 5)	(0, 0)	(0.04, 0.08)	0.122	0.162
2	(100, 100)	(0.04, 0.04)	(0.04, 0.08)	0.947	0.948
3	(5, 5)	(0, 0.04)	(0.04, 0.08)	0.112	0.149
4	(100, 100)	(0, 0.04)	(0.04, 0.08)	0.973	0.974
5	(5, 5)	(0, 0)	(0.08, 0.04)	0.121	0.165
6	(100, 100)	(0.04, 0.04)	(0.08, 0.04)	0.946	0.947
7	(5, 5)	(0, 0.04)	(0.08, 0.04)	0.101	0.129
8	(100, 100)	(0, 0.04)	(0.08, 0.04)	0.971	0.971
9	(100, 100, 100, 100)	(0.04, 0.04, 0.04, 0.04)	(0.04, 0.04, 0.04, 0.08)	0.976	0.977
10	(20, 70, 130, 180)	(0.04, 0.04, 0.04, 0.04)	(0.04, 0.04, 0.04, 0.08)	0.994	0.995
11	(100, 100, 100, 100)	(0, 0, 0.04, 0.04)	(0.04, 0.04, 0.04, 0.08)	0.982	0.983
12	(20, 70, 130, 180)	(0.08, 0.04, 0.04, 0)	(0.04, 0.04, 0.04, 0.08)	0.998	0.998
13	(20, 70, 130, 180)	(0, 0.04, 0.04, 0.08)	(0.04, 0.04, 0.04, 0.08)	0.981	0.983
14	(100, 100, 100, 100)	(0.04, 0.04, 0.04, 0.04)	(0.08, 0.04, 0.04, 0.04)	0.974	0.976
15	(20, 70, 130, 180)	(0.04, 0.04, 0.04, 0.04)	(0.08, 0.04, 0.04, 0.04)	0.502	0.521
16	(100, 100, 100, 100)	(0, 0, 0.04, 0.04)	(0.08, 0.04, 0.04, 0.04)	0.997	0.997
17	(20, 70, 130, 180)	(0.08, 0.04, 0.04, 0)	(0.08, 0.04, 0.04, 0.04)	0.417	0.434
18	(20, 70, 130, 180)	(0, 0.04, 0.04, 0.08)	(0.08, 0.04, 0.04, 0.04)	0.635	0.650
19	(36, 40, 16, 43, 72, 134)	Sampled from a [0, 0.04] uniform distribution per group	Sampled from a [0.04, 0.08] uniform distribution per group	0.570	0.602
20	(63, 58, 40, 31, 29, 25, 20, 19, 16, 10, 9, 9, 7, 5, 4, 3)	Sampled from a [0, 0.04] uniform distribution per group	Sampled from a [0.04, 0.08] uniform distribution per group	0.332	0.562

The power analysis shows that the less conservative asymptotic test has higher power. However, the difference in power is small, except for in very small sample sizes (simulations 1, 3, 5, 7) or with many unbalanced clusters (simulations 19 and 20) where the asymptotic test is especially non conservative.

Dataset sizes and number of events

Dataset name	Number of samples	Number of events
TCGA AML	170	103
TCGA BIC	621	79
TCGA COAD	220	50
TCGA GBM	274	218
TCGA KIRC	183	48
TCGA LIHC	367	127
TCGA LUSC	341	137
TCGA SKCM	448	212
TCGA OV	287	172
TCGA SARC	257	97
TCGA breast cancer used in Clusternomics	348	36
Murine analysis	16	12

More information on the TCGA datasets is available in Rappoport and Shamir (2018), and on the murine and Clusternomics datasets in Joachim et al (2018) and Gabasova et al (2017) respectively.

Detailed explanation of the analyses

P-values of the asymptotic vs. exact test on TCGA data.

Given each clustering solution, we computed its AP using survdiff function in the R package survival. We then computed its EP using our implementation of the ECF method from Heinze et al (2003). For each clustering solution, we first performed min(max(10 / AP, 1000), 1e5) iterations, and then repeatedly performed additional 1e4 iterations until both the low and high bounds of the 95% confidence interval were 10% the estimated p-value, and the confidence interval did not intersect the significance threshold of 0.05, up to a maximum of 1e7 iterations. These results are presented in **Figure 1A**.

MCCA permutation analysis.

Given the clustering solution of MCCA on Kidney Renal Clear Cell Carcinoma from TCGA, we performed one million permutations and imputed follow-up and survival times as described in Heinze et al (2003). For each such permutation, we calculated AP using the survdiff function from the R survival library. These p-values are presented as a histogram in **Figure 1B**. The fraction of times where the asymptotic p-value was ≤ 0.05 was 10.9%.

We also present the Kaplan-Meier curve for the clustering solution (**Appendix Figure S1**). The large difference in survival is largely due to a small cluster with very early events.

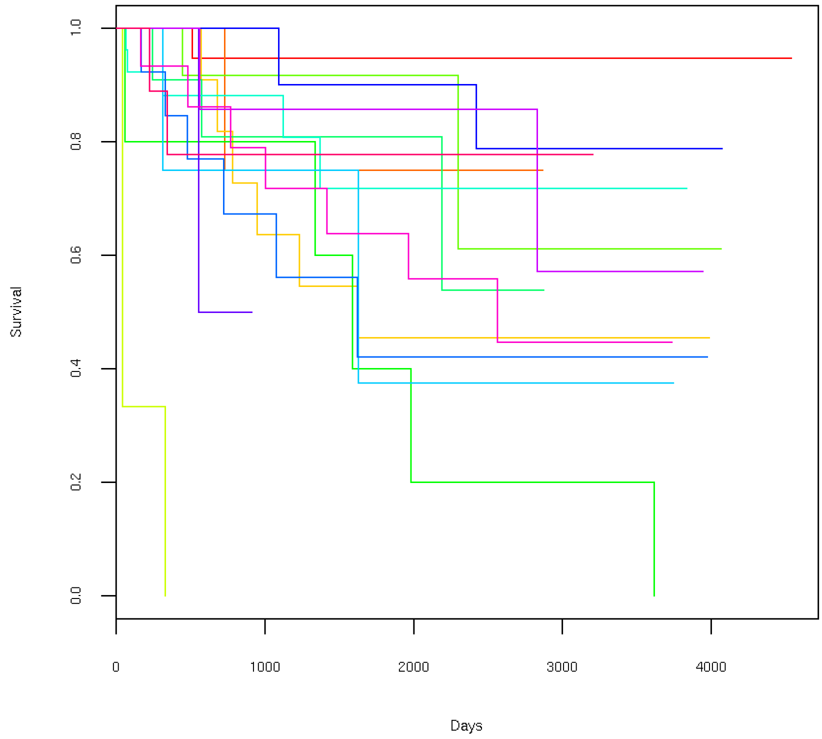

Appendix Figure S1: Kaplan-Meier curve for the clustering of MCCA on the Kidney Renal Clear Cell Carcinoma from TCGA.

Breast cancer analysis.

For each number of clusters k between 2 and 20, we clustered 621 Breast cancer patients from TCGA randomly into k-1 clusters with 10 patients, and one cluster with the rest of the patients. For each k, given these cluster sizes, we randomly permuted the patients' cluster labels (without imputing the survival and follow-up times, since we do not compute the p-value for a specific grouping). For each permutation we calculated the AP, and for each k estimated the fraction of permutations with AP≤ 0.05. We performed permutations until 0.05 was not within the 95% confidence interval and its lower and higher bounds were within 10% of the estimated AP, and up to a maximum of 5 million permutation. These results are presented in **Figure 1C**.

Children sepsis mortality

The data used for this test were extracted from Figure 3 in Joachim et al (2018). They are available on this project's Github page.

Since the data do not contain censored samples, the permutational log-rank test is equivalent to the conditional test. We therefore performed an exact permutational test, using the function logrank_test from the package coin, with parameter distribution='exact'. Since these data contain events with identical times, we executed several ways to handle the tied times. We used the logrank_test method with three different parameters for the "ties.method" argument: 'mid-ranks', 'average-scores' and 'Hothorn-Lausen'. The reported

p-values from these runs are 0.0597, 0.0559 and 0.1166, all of which are not significant using the threshold of 0.05 used in the original publication.

Clusternomics

The data were available as part of the clusternomics R package (Gabasova et al, 2017): https://cran.r-project.org/web/packages/clusternomics/index.html

The authors reported obtaining a p-value of 0.038 for the data, but did not publish their clustering solution. They also did not state the version of the log-rank test they use. As stated previously, in retrospective analysis of cancer data, different groups of patients can have different follow-up functions.

To assess the relevance of reporting the AP to show the clustering's merit, we permuted the data multiple times and for each permutation of the class labels computed the AP. We estimated the fraction of permutations for which the p-value was ≤ 0.038, and repeated the permutations using the same criteria as in the analysis from Figure 1C. The obtained estimate was 0.135.
Again, we stress that this permutation testing is not equivalent to the conditional log-rank test, and does not show that the p-value reported by the authors is incorrect. However, this analysis shows that reporting the AP does not suffice to convincingly show a clustering's advantage in such settings.

References

Brown M (1984). On the choice of variance for the log rank test. *Biometrika*, 71(1):65–74.

Gabasova E, Reid J, Wernisch L (2017) Clusternomics: Integrative context-dependent clustering for heterogeneous datasets. *PLOS Comput Biol* 13, 10, e1005781.

Heinze G, Gnant M, Schemper M (2003) Exact log-rank tests for unequal follow-up. *Biometrics* 59, 4, 1151–1157.

Joachim RB, Altschuler GM, Hutchinson JN, Wong HR, Hide WA, Kobzik L (2018) The relative resistance of children to sepsis mortality: from pathways to drug candidates. *Mol Syst Biol* 14, 5.

Mantel, N (1966) Evaluation of survival data and two new rank order statistics arising in its consideration. *Cancer Chemother Rep* 50, 163– 170.

Mantel N (1985). Propriety of the Mantel-Haenszel variance for the log rank test. *Biometrika*, 72(2):471–2.

Peto R, Peto J (1972) Asymptotically efficient rank invariant procedures (with discussion). *J R Stat Soc Series A*, 135, 185– 206.

Rappoport N, Shamir R (2018) Multi-omic and multi-view clustering algorithms: review and cancer benchmark. *Nucleic Acids Res*, gky889.

Vandin F, Papoutsaki A, Raphael BJ, Upfal E (2015) Accurate Computation of Survival Statistics in Genome-Wide Studies. *PLOS Comput Biol* 11(5): e1004071.

Wang R, Lagakos SW, Gray RJ (2010) Testing and interval estimation for two-sample survival comparisons with small sample sizes and unequal censoring. *Biostatistics* 11, 4 (05), 676–692.
